# Gestational Diabetes Mellitus Management Among Primigravida Women: A Qualitative Study

**DOI:** 10.7759/cureus.88710

**Published:** 2025-07-24

**Authors:** Kalyani Rath, Moonjelly Vijayan Smitha

**Affiliations:** 1 Obstetrics and Gynaecology, Kalinga Institute of Nursing Sciences, Bhubaneswar, IND; 2 Obstetrics and Gynecology, College of Nursing, All India Institute of Medical Sciences, Bhubaneswar, Bhubaneswar, IND

**Keywords:** emotional response, gestational diabetes mellitus, primigravida women, qualitative study, self-management

## Abstract

Background: Gestational diabetes mellitus (GDM) is a growing public health concern, particularly in India, which has a high prevalence of diabetes. While much research focuses on clinical outcomes, there is limited understanding of the lived experiences of primigravida women diagnosed with GDM.

Objective: This qualitative study aimed to explore the emotional responses, self-management practices, support systems, and healthcare interactions of first-time pregnant women diagnosed with GDM.

Methods: Using a phenomenological approach, 18 primigravida women with GDM attending a tertiary care hospital in Bhubaneswar, India, were recruited as a sample through purposive sampling. Semi-structured interviews were conducted in Odia, audio-recorded, transcribed verbatim, and thematically analyzed with NVivo software.

Results: Six major themes emerged during the course of the study. The emotional responses included shock, anxiety, fear for the baby’s health, and self-blame. Lifestyle modifications were reported as challenging, especially dietary restrictions conflicting with cultural food habits, glucose monitoring discomfort, and difficulty maintaining physical activity. Family and peer support were vital for coping, while a lack of community awareness led to stigma and isolation. Healthcare interactions varied, with clear communication and empathy fostering trust, but inconsistent advice causing confusion. Knowledge empowerment and maternal motivation enhanced self-efficacy and adaptive routines. Concerns about future diabetes risk and GDM recurrence were common, with inadequate postpartum counseling noted.

Conclusion: Primigravida women with GDM face complex emotional, cultural, and practical challenges. Culturally tailored dietary guidance, empathetic healthcare communication, strengthened social support, and extended postpartum care are essential to improve self-management and health outcomes. Addressing these aspects holistically can enhance care quality and reduce diabetes burden in diverse settings.

## Introduction

Diabetes is primarily a lifestyle condition that has increased alarmingly across all age groups in India, and the prevalence among the younger population has increased above 10%. India is now referred to as the “diabetes capital of the world” [1], as it accounts for 17% of the total number of global diabetes patients. Diabetes is a significant global health issue, with currently close to 80 million people with diabetes in India, and this number is expected to increase to 135 million by 2045 [2].

Gestational diabetes mellitus (GDM) is defined as a form of hyperglycemia or glucose intolerance that is first recognized or diagnosed during pregnancy. It typically manifests as elevated blood glucose levels that do not meet the criteria for overt diabetes but are above normal pregnancy glucose thresholds [3].

The prevalence of GDM is increasing, driven by rising obesity rates, sedentary lifestyles, and advanced maternal age. The International Diabetes Federation (IDF) reported that in 2019, around 23 million women aged 20-79 were living with diabetes, including GDM, with projections reaching 343 million by 2045. In 2021, an estimated 21.1 million live births were affected by hyperglycemia in pregnancy, 80.3% of which were due to GDM [4]. In particular, hyperglycaemia in pregnancy affects 20 million women annually, which accounts for 16% of all live births, with 84% of these cases attributed to gestational diabetes. Globally, one in six births is affected by gestational diabetes, highlighting the scale of the issue [5].

GDM is associated with a spectrum of adverse outcomes, including macrosomia, birth trauma, preeclampsia, increased caesarean section rates, and neonatal complications such as hypoglycaemia and respiratory distress syndrome [6]. Long-term risks include the development of type 2 diabetes mellitus and cardiovascular diseases in both the mother and the offspring [7]. Effective GDM management typically involves lifestyle modifications, including dietary changes, physical activity, regular blood glucose monitoring, and pharmacological intervention when necessary [8]. However, adherence to these recommendations is often challenging, particularly for primigravida women who are also adjusting to the emotional and physical demands of first-time pregnancy.

Primigravida women may experience heightened vulnerability when diagnosed with GDM, as they are less familiar with pregnancy-related changes and the healthcare system [9]. The diagnosis can trigger emotional responses such as anxiety, fear, guilt, and confusion. Social and cultural beliefs, family dynamics, and healthcare communication further influence how women perceive and manage their condition [10]. Moreover, the transition from being a healthy pregnant woman to being medically monitored can alter their perception of pregnancy and self-identity.

While numerous quantitative studies have explored the epidemiology and clinical outcomes of GDM, there is a paucity of qualitative research focusing on the lived experiences of affected women, particularly first-time mothers. Understanding primigravida women's experiences and managing GDM is essential for culturally sensitive interventions and enhancing patient-centred care. Such insights are particularly valuable in low- and middle-income countries where health literacy, resource access, and cultural expectations vary widely.

This qualitative study aims to explore the experiences of primigravida women diagnosed with GDM, focusing on their emotional responses, self-management practices, support systems, and interactions with healthcare providers within the local cultural and socioeconomic context. The findings are intended to inform healthcare strategies that better support women in navigating GDM during their first pregnancy.

## Materials and methods

Study design

This study employed a qualitative, phenomenological research design to explore the lived experiences of primigravida women in managing GDM. The phenomenological approach was adopted to capture the depth and complexity of personal experiences, perceptions, and coping strategies related to GDM management during first-time pregnancies.

Sample and sampling strategy

The study was conducted at the outpatient department of Obstetrics and Gynaecology between January and March 2025, among 18 women diagnosed with GDM at Pradyumna Bal Memorial Hospital (PBMH), Kalinga Institute of Medical Sciences (KIMS), a tertiary care center in Bhubaneswar, Odisha, India. A purposive sampling technique was used to recruit the participants from the population who met the predetermined inclusion criteria. The sampling criteria included consenting women diagnosed with gestational diabetes attending the outpatient department of the study center. Those with a prior diagnosis of chronic conditions such as cardiovascular disease, kidney disease, previous hypertension, or pre-existing diabetes were excluded.

Data collection

An interview guide was developed based on existing literature and expert consultation, and included open-ended questions focusing on participants’ understanding of GDM, emotional responses, lifestyle modifications, healthcare interactions, and social support. Each interview lasted approximately 30 to 40 minutes and was audio-recorded with the participants' informed consent. Data collection was carried out after obtaining IEC approval and permission from the medical superintendent of the institution. The purpose of the study was clearly articulated after obtaining verbal consent from the participating women. Data were collected through in-depth, semi-structured interviews conducted in March in the examination room near the antenatal OPD. A total of 18 primigravida women participated in the study. All interviews were conducted in the Odia language to ensure clarity and ease of communication. The researcher approached the participants when they were physically and emotionally comfortable. The approximate duration of data collection was communicated to them. The researcher expressed gratitude to the women who took the time for data collection. All interviews were transcribed verbatim and anonymized to ensure confidentiality.

Data analysis

Thematic analysis was conducted. The analysis began with immersion in the data through repeated readings of the transcripts to ensure comprehensive familiarization. This was followed by systematic coding to identify significant features across the dataset. Related codes were systematically clustered and organized into preliminary themes, capturing salient patterns that encapsulated participants’ experiences and perceptions in a coherent and meaningful manner. NVivo qualitative data analysis software, version 15, trial version (Lumivero, Denver, CO), was utilized to support the organization, coding, and retrieval of data, enhancing the rigor and transparency of the analytic process.

Ethical considerations

The institutional ethical committee ethically approved this study vide reference Number KIIT/KIMS/IEC/918/2022. Informed consent was obtained from mothers before the collection of the data. Confidentiality and anonymity were maintained in the recording and storage of data throughout the study.

Trustworthiness of the study

Credibility was enhanced through prolonged engagement with participants, iterative data analysis, and member checking, allowing participants to validate the interpretations of their narratives. Transferability was supported by providing rich, contextual descriptions of the study setting, participant demographics, and data collection processes, enabling readers to assess applicability to other contexts. Dependability was ensured through comprehensive documentation of the research design, data collection procedures, and analytical decisions.

## Results

This study included 18 primigravida women diagnosed with GDM who participated in in-depth interviews. Participants ranged from 22 to 34 years of age and were in their second or third trimester. All were purposively selected to ensure variation in age, socio-economic background, and healthcare access. Thematic analysis revealed six primary themes with interconnected sub-themes, illustrating a holistic view of their lived experiences.

Theme: Emotional response to diagnosis

Sub-theme: Initial Shock and Anxiety

The GDM diagnosis was described by many as a turning point in the pregnancy experience. Several women reported experiencing shock, confusion, and emotional distress, especially because this was their first pregnancy and they had limited prior knowledge of GDM.

"It was just a routine test, and suddenly they said I had diabetes. I was completely shocked. I didn’t even know what it meant." (P3)

The abrupt shift from a "normal" to a "high-risk" pregnancy identity generated anxiety and disrupted the anticipated joyful experience of early motherhood.

Sub-theme: Fear for Baby’s Health

A primary concern expressed was for the health and safety of the unborn child. Participants feared complications such as macrosomia, preterm labor, or stillbirth. This fear often overshadowed their own health concerns.

"I don’t care about me-I keep thinking, what if my baby is born unhealthy? That’s what scared me the most." (P7)

Sub-theme: Sense of Guilt or Self-blame

Several participants internalized the diagnosis and perceived it as a personal failure. These feelings were often rooted in misunderstandings about the causes of GDM.

"I kept asking myself, did I eat too many sweets? Was it because I didn’t exercise? I blamed myself a lot." (P8)

This self-blame occasionally led to shame and isolation, particularly when participants felt judged by family or community.

Theme: Challenges in adapting to lifestyle modifications

Sub-theme: Dietary Restrictions and Cultural Conflicts

Managing diet was one of the most significant and challenging aspects of GDM care. Many women found it difficult to align dietary recommendations with traditional food practices.

"In our culture, rice is in every meal. Cutting that out felt like cutting a part of our life out." (P7)

Sub-theme: Struggles With Routine Glucose Monitoring

Participants described glucose monitoring as a physically uncomfortable and emotionally intrusive routine. Some expressed distress at the medicalization of pregnancy and the constant reminders of their diagnosis.

"Pricking my finger multiple times a day just made me feel like a patient, not a pregnant woman enjoying her journey." (P13)

Sub-theme: Managing Physical Activity During Pregnancy

Although participants were advised to engage in light physical activity, most reported difficulties in sustaining routines due to fatigue, pregnancy-related discomfort, and fear of harming the baby.

"I wanted to go walking, but sometimes I was just too tired or scared it would hurt the baby." (P5)

Some lacked access to safe or supportive environments for exercise, especially those living in urban or densely populated areas.

Theme: Support system and its impact

Sub-theme: Role of Partner and Family Support

Support from spouses and family members played a critical role in shaping participants’ ability to cope with the diagnosis. Active involvement from family, like preparing meals or accompanying women on walks, greatly eased the management burden.

"My mother changed her cooking just for me. My husband walked with me every evening. I felt I wasn’t alone." (P2)

Sub-theme: Isolation Due to Lack of Understanding

A lack of awareness about GDM within the community contributed to stigma and emotional isolation. Participants noted that their symptoms were sometimes dismissed or minimized.

"They said it was just 'pregnancy sugar' and not a real problem. I felt like nobody understood." (P14)

This lack of validation led some to conceal their diagnosis or avoid discussing it altogether.

Theme: Interactions with healthcare providers

Sub-theme: Clarity and Communication of Information

Clear, simple explanations from healthcare providers were highly valued. Participants who received detailed and empathetic communication felt more confident in their ability to manage the condition.

"The doctor showed me pictures and explained everything in my language. That made me feel safe and informed." (P1)

Sub-theme: Perceived Empathy

The perceived emotional support from healthcare professionals influenced how participants viewed the entire healthcare system. Empathy built trust; a lack of it left women feeling dismissed.

"Some doctors just rushed me. But one midwife sat down and asked how I was coping--that meant the world to me." (P6)

Theme: Self-efficacy and coping strategies

Sub-theme: Empowerment through Knowledge

Participants who took the initiative to learn about GDM reported a greater sense of control. Gaining knowledge reduced anxiety and motivated them to comply with management strategies.

"Once I understood what insulin resistance meant, I realized I could take control of it." (P12)

Sub-theme: Personal Motivation for Baby’s Well-being

The unborn child served as a powerful emotional motivator. Many women made lifestyle changes they might not have attempted otherwise because of their deep desire to protect their baby.

"Every craving I ignored, every walk I took--it was all for my baby. That gave me strength." (P4)

Sub-theme: Adaptive Routines and Time Management

Creating personalized routines helped participants manage the demands of diet, testing, and appointments without feeling overwhelmed.

"I wrote everything down-what I ate, when I walked, sugar levels. It became a part of my day." (P9)

Theme: Long-term health concerns and future outlook

Sub-theme: Fear of Type 2 Diabetes Post-delivery

There was widespread anxiety about developing Type 2 diabetes later in life. Many participants had heard that GDM increased the risk, but few received formal counseling on prevention.

"They told me I could get diabetes later, but no one told me how to avoid it. That’s scary." (P15)

Sub-theme: Motivation for Sustained Lifestyle Changes

For some, the diagnosis served as a wake-up call. These participants viewed GDM as a turning point for positive, long-term health behaviors.

"After this, I started eating better and walking every day--not just for the pregnancy, but for myself." (P18)

## Discussion

This qualitative study examined the lived experiences of first-time mothers managing GDM. It highlighted the complex interactions among emotional, social, cultural, and healthcare-related factors. The findings provide a detailed understanding of how primigravida women perceive, adapt to, and cope with a GDM diagnosis during pregnancy.

Consistent with prior research, the initial emotional response to GDM included shock, fear, and anxiety, often due to limited knowledge and a sudden reclassification of pregnancy as "high-risk" [11,12]. For primigravida women, who may expect pregnancy to be joyful, the diagnosis signified a disorienting shift. The guilt and self-blame reported by participants reflect a lack of awareness about GDM's multifactorial causes and social messages that unfairly burden maternal behavior [13].

Lifestyle modification challenges, particularly dietary restrictions, emerged as a prominent theme. Participants reported cultural conflicts, with traditional diets often centered around carbohydrate-rich staples like rice. This aligns with findings from studies conducted in other South Asian and low-resource contexts, where culturally insensitive dietary advice contributed to confusion and non-adherence [10,14]. Glucose monitoring and physical activity were also perceived as burdensome, especially when compounded by pregnancy fatigue, lack of safe spaces for exercise, or insufficient financial resources. These challenges underline the need for context-sensitive, individualized care plans that accommodate cultural food practices and socioeconomic realities.

The role of social support is pivotal. As observed in other studies, emotional and practical support from family, especially spouses and mothers, played a critical role in enhancing self-management capacity and reducing psychological stress [15]. In contrast, women who lacked understanding from family or community members reported emotional isolation and stigma.

Participants’ interactions with healthcare providers revealed varying degrees of satisfaction. While some reported receiving clear, empathetic communication. These findings reinforce the importance of continuity of care, effective communication, and provider empathy in fostering trust and adherence to medical guidance [16].

A key strength among many participants was their emerging self-efficacy. Knowledge acquisition and a strong maternal drive to protect the unborn child served as powerful motivators for compliance. Similar to findings in previous studies, women who understood the rationale behind lifestyle changes were more likely to adopt adaptive routines and persist with self-care behaviors [17]. This highlights the critical role of targeted health education and empowerment in GDM management.

Participants expressed apprehension regarding potential long-term health risks, such as the progression to type 2 diabetes and the recurrence of GDM in subsequent pregnancies. Although their concerns were legitimate, the absence of formal postnatal counseling or follow-up contributed to a prevailing sense of uncertainty. This gap indicates a necessity to extend the care for GDM beyond the delivery phase, by incorporating postpartum education, diabetes prevention strategies, and routine screening within maternal health services [18].

Strengths and limitations of the study

A significant strength of this study lies in its focus on primigravida women, a demographic frequently neglected in research on GDM. The purposive sampling strategy employed ensured a rich diversity concerning age, socioeconomic status, and access to healthcare, thereby enhancing the data quality. Nevertheless, the findings may lack generalizability beyond contexts that are similar to urban and semi-urban areas in India. Furthermore, there may have been an influence of social desirability bias on the participants’ narratives during the interviews. Future research could investigate the perspectives of multiparous women or incorporate longitudinal follow-up to evaluate how experiences change postpartum.

Implications for practice

Enhancing the quality of care for individuals with GDM necessitates the integration of culturally sensitive dietary counseling, supported by the dissemination of simplified educational materials in local languages to improve accessibility and health literacy. Establishing structured emotional and peer support systems, such as facilitated support groups or digital health applications, can contribute significantly to psychological well-being by fostering a sense of community and shared experience. Strengthening healthcare provider training in empathetic communication and cultural competence is critical to fostering trust, improving patient-provider interactions, and enhancing adherence to therapeutic recommendations. Moreover, a comprehensive approach to postpartum care should encompass routine screening for type 2 diabetes, individualized counseling on recurrence risks in subsequent pregnancies, and sustained lifestyle interventions to mitigate long-term metabolic complications.

## Conclusions

This study emphasizes the complex challenges confronted by primigravida women diagnosed with GDM, which include emotional distress, difficulties in cultural and lifestyle adaptation, and varying experiences with healthcare support. The findings emphasize the necessity of providing culturally sensitive, patient-centered care that recognizes the unique needs of first-time mothers navigating GDM. Enhancing social support systems, fostering empathetic communication by healthcare providers, and ensuring comprehensive postpartum follow-up are vital for improving adherence, psychological well-being, and long-term health outcomes. Tailored interventions that empower women through education and peer connections can promote effective self-management and reduce future diabetes risks. Addressing these aspects holistically is essential for optimizing care and supporting positive maternal and neonatal health across diverse socio-cultural contexts.

## References

[1] (2024). The Economic Times. India has over 100 mn diabetics, 136 mn pre-diabetics, says new ICMR study. Times.

[2] (2024). The Times of India. Why India is diabetes capital of the world. https://timesofindia.indiatimes.com/india/why-india-is-diabetes-capital-of-the-world/articleshow/95509990.cms.

[3] American Diabetes Association (2004). Gestational diabetes mellitus. Diabetes Care.

[4] Wang H, Li N, Chivese T (2022). IDF Diabetes Atlas: Estimation of global and regional gestational diabetes mellitus prevalence for 2021 by International Association of Diabetes in Pregnancy Study Group's criteria. Diabetes Res Clin Pract.

[5] Akinyemi OA, Weldeslase TA, Odusanya E (2023). Profiles and outcomes of women with gestational diabetes mellitus in the United States. Cureus.

[6] Farrar D, Simmonds M, Bryant M (2016). Treatments for gestational diabetes: a systematic review and meta-analysis. BMJ.

[7] Bellamy L, Casas JP, Hingorani AD, Williams D (2009). Type 2 diabetes mellitus after gestational diabetes: a systematic review and meta-analysis. Lancet.

[8] Metzger BE, Gabbe SG, Persson B (2010). International association of diabetes and pregnancy study groups recommendations on the diagnosis and classification of hyperglycemia in pregnancy. Diabetes Care.

[9] Sohmaran C, Mohamed Rahim AB, Chua JYX, Shorey S (2023). Perceptions of primiparous women diagnosed with gestational diabetes mellitus: A descriptive qualitative study. Midwifery.

[10] Bandyopadhyay M, Small R, Davey MA, Oats JJ, Forster DA, Aylward A (2011). Lived experience of gestational diabetes mellitus among immigrant South Asian women in Australia. Aust N Z J Obstet Gynaecol.

[11] Carolan M, Gill GK, Steele C (2012). Women's experiences of factors that facilitate or inhibit gestational diabetes self-management. BMC Pregnancy Childbirth.

[12] Lapolla A, Dalfrà MG, Fedele D (2009). Management of gestational diabetes mellitus. Diabetes Metab Syndr Obes.

[13] Craig L, Sims R, Glasziou P, Thomas R (2020). Women's experiences of a diagnosis of gestational diabetes mellitus: a systematic review. BMC Pregnancy Childbirth.

[14] Kim C, McEwen LN, Kieffer EC, Herman WH, Piette JD (2008). Self-efficacy, social support, and associations with physical activity and body mass index among women with histories of gestational diabetes mellitus. Diabetes Educ.

[15] Hjelm K, Bard K, Nyberg P, Apelqvist J (2007). Management of gestational diabetes from the patient's perspective--a comparison of Swedish and Middle-Eastern born women. J Clin Nurs.

[16] Holmes-Walker DJ, Llewellyn AC, Farrell K (2007). A transition care programme which improves diabetes control and reduces hospital admission rates in young adults with Type 1 diabetes aged 15-25 years. Diabet Med.

[17] Morrison MK, Lowe JM, Collins CE (2014). Australian women's experiences of living with gestational diabetes. Women Birth.

[18] Ferrara A (2007). Increasing prevalence of gestational diabetes mellitus: a public health perspective. Diabetes Care.

